# Resting‐state connectivity predicts levodopa‐induced dyskinesias in Parkinson's disease

**DOI:** 10.1002/mds.26540

**Published:** 2016-03-08

**Authors:** Damian M. Herz, Brian N. Haagensen, Silas H. Nielsen, Kristoffer H. Madsen, Annemette Løkkegaard, Hartwig R. Siebner

**Affiliations:** ^1^ Danish Research Centre for Magnetic Resonance, Centre for Functional and Diagnostic Imaging and ResearchCopenhagen University Hospital HvidovreHvidovreDenmark; ^2^ Medical Research Council Brain Network Dynamics Unit at the University of OxfordOxfordUnited Kingdom; ^3^ Nuffield Department of Clinical NeurosciencesUniversity of Oxford, John Radcliffe HospitalOxfordUnited Kingdom; ^4^ Department of NeurologyCopenhagen University Hospital BispebjergCopenhagenDenmark; ^5^ Cognitive Systems, Department of Applied Mathematics and Computer ScienceTechnical University of DenmarkKgs. LyngbyDenmark

**Keywords:** dyskinesias, fMRI, levodopa MRI, Parkinson's disease

## Abstract

**Background:**

Levodopa‐induced dyskinesias are a common side effect of dopaminergic therapy in PD, but their neural correlates remain poorly understood.

**Objectives:**

This study examines whether dyskinesias are associated with abnormal dopaminergic modulation of resting‐state cortico‐striatal connectivity.

**Methods:**

Twelve PD patients with peak‐of‐dose dyskinesias and 12 patients without dyskinesias were withdrawn from dopaminergic medication. All patients received a single dose of fast‐acting soluble levodopa and then underwent resting‐state functional magnetic resonance imaging before any dyskinesias emerged. Levodopa‐induced modulation of cortico‐striatal resting‐state connectivity was assessed between the putamen and the following 3 cortical regions of interest: supplementary motor area, primary sensorimotor cortex, and right inferior frontal gyrus. These functional connectivity measures were entered into a linear support vector classifier to predict whether an individual patient would develop dyskinesias after levodopa intake. Linear regression analysis was applied to test which connectivity measures would predict dyskinesia severity.

**Results:**

Dopaminergic modulation of resting‐state connectivity between the putamen and primary sensorimotor cortex in the most affected hemisphere predicted whether patients would develop dyskinesias with a specificity of 100% and a sensitivity of 91% (*P* < .0001). Modulation of resting‐state connectivity between the supplementary motor area and putamen predicted interindividual differences in dyskinesia severity (*R* ^2^ = 0.627, *P* = .004). Resting‐state connectivity between the right inferior frontal gyrus and putamen neither predicted dyskinesia status nor dyskinesia severity.

**Conclusions:**

The results corroborate the notion that altered dopaminergic modulation of cortico‐striatal connectivity plays a key role in the pathophysiology of dyskinesias in PD. © 2016 International Parkinson and Movement Disorder Society

## Introduction

Levodopa‐induced dyskinesias (LID) are a common and disabling side effect of levodopa treatment of PD. The main risk factors are disease duration, levodopa dose, and age at onset.^1^, ^2^ Yet these factors alone cannot predict whether an individual patient will develop LID. Functional magnetic resonance imaging (fMRI) is a widely used method to study disease‐related changes in functional connectivity while patients are at rest and without engaging in a specific task.^3^, ^4^ In PD, fMRI‐based measures of resting‐state connectivity can distinguish patients from healthy controls.^5^ However, it remains unknown whether resting‐state fMRI can identify patients at risk to develop LID or assess the antidyskinetic effects of novel drugs. Although animal studies have provided strong evidence for a central role of the putamen and its cortical projections in LID,^6^, ^7^ recent MRI studies have additionally identified cortical regions playing key roles in the development of LID comprising the supplementary motor area (SMA),^8^, ^9^, ^10^ primary sensorimotor cortex (SM_1_),^9^, ^11^ and right inferior frontal gyrus (rIFG).^8^, ^11^, ^12^ In this study, we employed fMRI to study acute levodopa‐induced changes in cortico‐striatal resting‐state connectivity in patients with and without peak‐of‐dose LID. We hypothesized that patients with LID would show an altered levodopa‐induced modulation of cortico‐striatal connectivity that would predict the emergence and severity of LID. Based on previous studies,^12^, ^13^ we also acquired structural MRI to assess whether structural changes of these regions would predict LID.

## Methods

### Participants

A total of 40 patients with a clinical diagnosis of PD were enrolled in the study. Exclusion criteria comprised (i) tremor‐dominant PD, (ii) psychiatric illness, (iii) dementia, (iv) contraindications to MRI, (v) sedatives or serotonergic medication, and (vi) off‐ or biphasic dyskinesias. Ten patients were excluded because they did not tolerate withdrawal of medication or developed claustrophobia inside the scanner. Fourteen of the remaining 30 patients had choreiform peak‐of‐dose dyskinesias (LID group). All 14 LID patients underwent structural T1‐weighted MRI of the brain. Two of the 14 patients had to be excluded from the analysis of resting‐state fMRI because they developed dyskinesias before the first postlevodopa resting‐state fMRI scan (see below). A total of 10 PD patients without LID (No‐LID) participated in both structural MRI and resting‐state fMRI. Of the No‐LID patients, 4 underwent only structural MRI, and in 2 No‐LID patients only resting‐state fMRI could be acquired due to technical problems.

The LID and No‐LID group were matched regarding multiple clinical measures (Table 1). Levodopa‐equivalent daily dose^14^ was higher in the LID patients (resting‐state fMRI study: *P*_uncorrected_ = .031, structural MRI study: *P*_uncorrected_ = .045). We did not assess blood levels of levodopa because previous studies have shown no differences in the pharmacokinetics of levodopa between patients with and without LID.^15^, ^16^ The study was performed in accordance with the declaration of Helsinki. All patients gave informed consent to participating in the study, which was approved by the ethics committee of the Capital Region of Denmark (study numbers: H‐2‐2010‐146 and H‐3‐2011‐110).

**Table 1:** Overview of clinical and demographic characteristics

	Resting‐state fMRI study			Structural MRI study		
	LID (n = 12)	No‐LID (n = 12)	*P*	LID (n = 14)	No‐LID (n = 14)	*P*
Gender	6f	4f	>.5	7f	5f	>.5
Handedness	10r	11r	>.5	12r	12r	>.5
Age (y)	66.8 ± 9.1	67.3 ± 6.8	>.5	69.0 ± 10.0	64.2 ± 9.4	>.1
Education (y)	14.1 ± 4.1	13.9 ± 3.5	>.5	14.3 ± 4.0	13.2 ± 3.0	>.1
MMSE	28.9 ± 1.8	29.6 ± 0.9	>.1	28.9 ± 1.7	29.5 ± 0.9	>.1
MoCA	27.8 ± 2.8	28.6 ± 1.1	>.1	27.7 ± 2.6	28.7 ± 0.8	>.1
BIS‐11	59.3 ± 8.7	55.0 ± 7.7	>.1	58.6 ± 8.3	57.1 ± 9.5	>.5
Disease duration (y)	7.1 ± 3.8	5.9 ± 3.4	>.1	8.0 ± 4.4	5.9 ± 3.4	>.1
LEDD (all)	1014.9 ± 407	696.7 ± 252	.031^a^	958.9 ± 404	686.4 ± 268	.045^a^
Levodopa (mg)	848.9 ± 384	470.9 ± 228	.008^a^	810.9 ± 367	504.6 ± 288	.021^a^
LEDD (no levodopa)	166.0 ± 118	225.8 ± 138	>.1	148.0 ± 119	181.8 ± 131	>.1
UPDRS‐III‐OFF	32.2 ± 10.7	33.2 ± 6.9	>.5	33.1 ± 10.4	33.2 ± 6.8	>.5
UPDRS‐III‐ON	19.3 ± 7.1	21.3 ± 5.3	>.1	20.4 ± 7.4	22.3 ± 4.1	>.1
Δ UPDRS‐III	12.8 ± 5.2	11.9 ± 4	>.5	12.7 ± 4.8	10.9 ± 4.1	>.1
UDysRS (obj.)	14.2 ± 7.8	‐	‐	15.6 ± 8.3	‐	‐

Gender and handedness were compared using chi‐square tests. Age, education, MMSE, MOCA, Barratt Impulsiveness Scale (BIS), disease duration, levodopa equivalent daily dose (LEDD), and UPDRS were compared using independent‐sample *t* tests. obj., objective, f, female; r, right; y, years.

a *P*_uncorrected_ < .05.

### Study Design and MRI Acquisition

**Figure 1:**
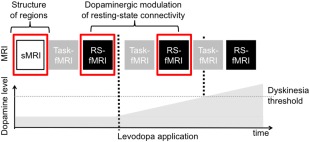
Study design. Structural scans (sMRI) were acquired in the off state. Resting‐state fMRI (RS‐fMRI) was recorded in the off‐medication state and repeatedly after levodopa intake (first vertical dashed line) until dyskinesias emerged (second vertical dashed line). [Color figure can be viewed in the online issue, which is available at wileyonlinelibrary.com.]

An MRI was acquired using a 3 T Verio scanner (Siemens, Erlangen, Germany) with a 32‐channel head coil. Prior to MRI acquisition, dopaminergic medication was withdrawn according to 6 half‐lives as described previously.^9^, ^10^ All MRI scans were acquired in the morning (see Fig. 1 for an overview). Initially, we recorded a T1‐weighted structural image of the whole brain (magnetization‐prepared rapid gradient echo (MPRAGE), field of view (FOV) 230 mm, slice thickness 0.9 mm, repetition time (TR) 1900 ms, echo time (TE) 2.32 ms, flip angle (FA) 9° sagittal orientation). We then acquired fMRI in the practical off state and subsequently after intake of 200 mg soluble levodopa + 50 mg benserazide dissolved in water (Madopar Quick, La Roche, Basel, Switzerland). We considered a 200‐mg dose of levodopa sufficient even in patients with long disease duration and high levodopa‐equivalent daily dose (LEDD) of their standard treatment because the clinical response to levodopa is more pronounced after a washout phase of dopaminergic medication than during standard treatment.^17^, ^18^ Post‐levodopa scans were acquired directly after intake of levodopa and before the emergence of dyskinesias while dopamine levels were gradually increasing.^10^ Scans were immediately stopped if a trained clinician (D.M.H.) inside the scanner room observed development of dyskinesias, and the respective fMRI session was discarded from the analysis. For example, if a patient developed LID in postlevodopa fMRI run 2, only the first postlevodopa fMRI run was used for analysis. This allowed us to assess dopaminergic modulation of resting‐state connectivity without confounding movement artifacts and behavioral differences between the LID and No‐LID group. This was particularly important because subtle dyskinesias or other involuntary movements could induce changes in resting‐state networks without necessarily inducing visible movement artifacts in the MR images. In case patients did not develop dyskinesias, scans were stopped after the second postlevodopa fMRI run. The fMRI measurements comprised interleaved runs of task‐related fMRI (∼9 min) and resting‐state fMRI (∼5 min). This design allowed us to control the framing of the resting state, that is, all patients performed the same simple motor task before and after acquisition of resting‐state fMRI runs. The procedures and results related to task‐related fMRI have been reported previously.^9^, ^10^ In this study, we significantly extend these previous reports by investigating whether differences in neural connectivity can distinguish patients with and without LID even in the absence of a motor task. An advantage of this would be that fMRI acquisition would be easier to standardize and apply in clinical settings. To prevent patients from falling asleep during the resting‐state fMRI, patients were instructed to remain still with eyes open while watching a gray screen. Each resting‐state fMRI session comprised 160 T2*‐weighted echo planar images (FOV 192 mm, slice thickness 3.5 mm, slice spacing 0.2 mm, TR 1850 ms, TE 26 ms, FA 75°, 36 slices with whole brain coverage).

### Data Analysis of Resting‐State fMRI

Analyses were conducted using Statistical Parametric Mapping (SPM 8 rev.4667, Wellcome Trust Centre for Neuroimaging, London, UK). Standard preprocessing procedures comprised accounting for T1‐equilibrium effects, realignment, correction for nuisance variables (respiration and cardiac pulsation, signals from white matter and cerebrospinal fluid, residual movement artefacts), normalization to stereotactic space using the Montreal Neurological Institute template, and smoothing with an isotropic Gaussian kernel at 8 mm full‐width at half maximum. The following steps were then conducted using a priori defined regions of interest (ROIs) using anatomical masks^19^ to avoid circularity when constraining the selected regions to the studied patient group.^20^ ROIs comprised the bilateral putamen and bilateral SM_1_ (BA 1‐4) as well as the SMA (bilateral BA 6) based on previous work.^9^, ^10^ The anatomical SMA mask comprised preSMA and SMA.^21^ In addition, we included rIFG based on recent work by Cerasa and colleagues on the neural correlates of LID.^8^, ^11^, ^12^ Because the previously reported rIFG coordinates varied along the anterior–posterior axis, we included both BA 44 and 45. For each patient, the time course of blood‐oxygen‐level dependent (BOLD) fluctuations from the right and left putamen were extracted using the first eigenvariate and used as regressors in separate general linear model (GLMs) applying a band‐pass filter between 0.008 and 0.1 Hz.^3^ Thus, the estimated beta weights reflected to what extent the BOLD time course in a given voxel covaried with the BOLD time course in the putamen constituting a measure of functional connectivity (covariance). Regional beta weights were extracted from (i and ii) the right and left SM_1_, (iii) the SMA, and (iv) the rIFG using the first eigenvariate from the unthresholded SPM of all voxels corresponding to the respective region.

For resting‐state connectivity between the SM_1_ and putamen, we extracted parameters from the most and less affected hemisphere, respectively, for each patient. Importantly, lateralization of predominant motor symptoms was equally distributed in the LID and No‐LID group (resting‐state fMRI: *P* > .5; structural MRI: *P* > .5, chi‐square tests). Because previously reported SMA activation in LID patients comprised a midline cluster extending into both hemispheres^8^, ^9^, ^10^ and IFG activation was mainly right lateralized,^8^, ^11^, ^13^ we refrained from extracting resting‐state connectivity values from the most and less affected hemisphere to avoid modeling interhemispheric connectivity (eg, left putamen to rIFG) in some patients and intrahemispheric connectivity (eg, right putamen to rIFG) in other patients. Instead, we used the right putamen for modeling rIFG–putamen connectivity and the mean of left and right putamen–SMA connectivity. Of note, connectivity parameters from these cortical ROIs to the left and right putamen, respectively, were highly correlated (*P* < .001, Pearson correlation). We computed dopaminergic modulation of resting‐state connectivity by subtracting the baseline measure in the off‐medication state from the postlevodopa measure. These parameters were transformed into *z* scores for further analyses (see below). Z‐scoring was done for each resting‐state connectivity measure (eg, SMA–putamen) for the whole group (LID and No‐LID patients together, n = 24) by subtracting the mean and dividing by the standard deviation, that is, it normalized the data to a mean of 0 and a standard deviation of 1. The results did not change when data were not z‐transformed (not shown).

To test whether regions outside the considered ROIs showed an abnormal levodopa‐induced modulation of connectivity with the putamen, we also conducted a 2 × 2 analysis of variance using Group (LID and No‐LID) and Dopamine (postlevodopa and OFF) as main effects and tested for a Group × Dopamine interaction. The results were thresholded at *P* = .05 family wise error (FWE)‐corrected at the cluster level using a cluster‐building threshold of *P*_uncorrected_ = 0.001.

### Data Analysis of Structural MRI

Cortical reconstruction and volumetric segmentation were performed with Freesurfer software (version 5.3; http://surfer.nmr.mgh.harvard.edu) using a standard processing pipeline. The technical details of these procedures have been previously described in detail.^22^, ^23^ In short, reconstruction procedures comprised intensity normalization to Montreal Neurological Institute (MNI)‐space, skull stripping, filtering, segmentation, and surface deformation. The quality of the skull stripping and accuracy of the gray and white matter outer boundaries were reviewed by a trained researcher (S.L.), who was blinded to the grouping of the patients. To extract the volumetric data of the predefined ROIs, we used specialized Freesurfer tools for automated parcellation of gray and white matter.^24^ As in the resting‐state fMRI analysis, ROIs comprised bilateral SM_1_ (BA 1‐4), SMA (bilateral BA 6), rIFG (BA 44 and 45), and bilateral putamen. The extracted volume measures were transformed into *z* scores for further analyses.

### Predictions of Emerging LID

**Figure 2:**
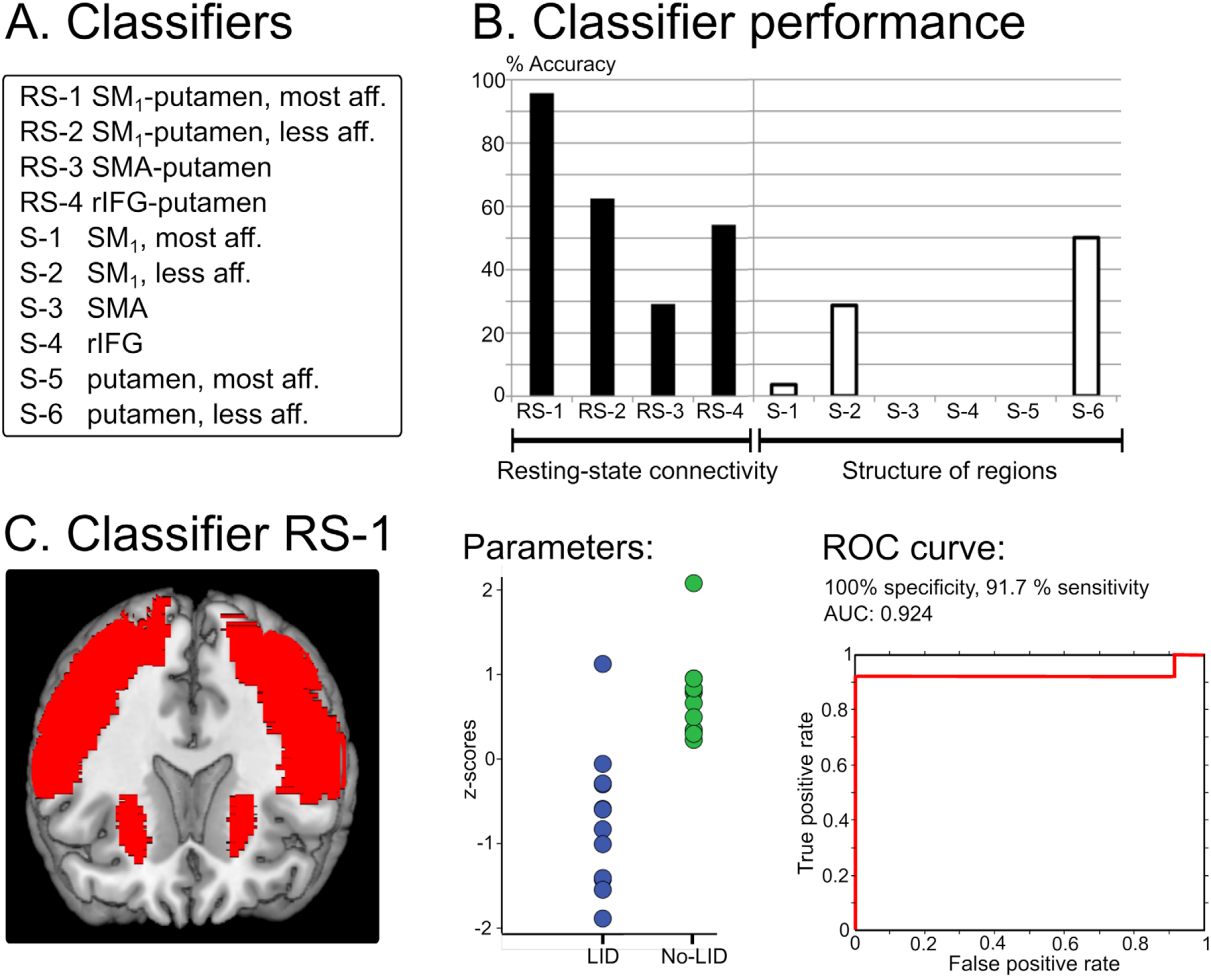
Classifier analyses. **A:** List of classifiers. **B:** Classifier accuracy in distinguishing patients with and without levodopa‐induced dyskinesias (LID). **C:** Key parameters of the best classifier (SM_1_‐putamen connectivity in most affected hemisphere). AUC, area under the curve; rIFG, right inferior frontal gyrus; ROC, receiver‐operating characteristic; RS, resting state; SM_1_, primary sensorimotor cortex; S, structural. [Color figure can be viewed in the online issue, which is available at wileyonlinelibrary.com.]

Measures reflecting dopaminergic modulation of resting‐state connectivity (n = 4) and gray matter volume (n = 6) were tested regarding their accuracy in classifying patients as LID or No‐LID patients (Fig. 2A). We applied a linear support vector classifier (C‐SVC; c‐value = 1) implemented in LIBSVM v3.17, www.csie.ntu.edu.tw/~cjlin/libsvm/ as described previously^10^ and used leave‐one‐out cross‐validation to compute classification accuracy, sensitivity and specificity, and the receiver operating characteristics (ROC) curve for computing the area under the curve (AUC). We used permutation tests (10,000 permutations) to derive the corresponding *P* values and applied Bonferroni‐correction for multiple comparisons. Additional classifier analyses considered resting‐state connectivity measures from the OFF and postlevodopa sessions separately.

### Predictions of LID severity

We applied linear regression analyses to test whether measures reflecting dopaminergic modulation of resting‐state connectivity predicted dyskinesia severity of the LID patients (ie, Unified Dyskinesia Rating Scale (UDysRS) scores as dependent variable). The Bonferroni method was applied to correct for multiple comparisons. One LID patient was detected as a significant outlier (Grubbs test) and thus excluded. Because patients with more severe LID also developed dyskinesias more quickly (*r* = –0.608, *P* = .036, Pearson correlation), we additionally conducted partial regression analyses using time to LID onset as a covariate to account for individual differences in the time between intake of levodopa and development of LID. We conducted the same regression analyses with gray matter volumes of ROIs. To assess whether putative predictions were specific for the development of dyskinesias, we repeated the analyses using the individual UPDRS OFF scores as a dependent variable.

### Additional Control Analyses

Because head movements can affect the analysis of resting‐state connectivity, we calculated the number of images moved more than 1 mm in relation to the previous image for each group and session.^10^ This analysis revealed that only 0.58% of images showed movement > 1 mm. Using this measure, head movements were not different between groups or sessions (Effect of Group, Session IA: *P* > .1). Furthermore, we measured respiration and pulsation during resting‐state fMRI acquisition because drug‐induced changes in these physiological parameters can affect measures of resting‐state connectivity.^25^ Mean respiration frequency was 14/min ± 4 (SD) and mean pulse rate was 72/min ± 14, which was not different between groups or sessions (Effect of Group, Session IA: *P* > .1).

## Results

All LID patients developed dyskinesias after an intake of 200 mg levodopa either inside the scanner room or shortly after completion of MRI acquisition. None of the No‐LID patients developed dyskinesias. In 7 LID patients, dyskinesias already emerged between the first and second postlevodopa resting‐state fMRI. Therefore, only the resting‐state fMRI scan in the off state and the first postlevodopa resting‐state fMRI were used for analysis of resting‐state connectivity because they were available in all participants. The first postlevodopa resting‐state fMRI was acquired approximately 25 min after levodopa intake (LID 23 ± 3 min vs. No‐LID 25 ± 5 min, *P* >.1; independent‐sample *t* tests).

Dopaminergic modulation of resting‐state connectivity between the SM_1_ and putamen in the most affected hemisphere (classifier RS‐1) significantly predicted whether an individual patient would develop dyskinesias (*P* < .0001; Fig. 2B). Although resting‐state connectivity (both *z‐*scored and non‐*z*‐scored covariance estimates) between SM_1_ and the putamen increased after levodopa intake in No‐LID patients, it decreased in LID patients. The classifier yielded 95.8% accuracy corresponding to 1 false negative prediction (91.7% sensitivity) and no false positive predictions (100% specificity), which is illustrated in Figure 2C. The other classifiers did not significantly differentiate between patients with and without LID, and no regions outside the considered ROIs showed a significant Group × Dopamine interaction effect. Furthermore, volumetric MRI measures of the ROIs did not significantly predict whether a patient would develop LID. For a detailed description of the performance of all classifiers, please see Table 2. Additional classifier analyses, which only considered the OFF or postlevodopa fMRI session, revealed that SM_1_–putamen resting‐state connectivity in the most affected hemisphere before intake of levodopa (OFF) predicted LID with an accuracy of 70.9% (83.3% sensitivity, 58.3% specificity), but this result did not survive Bonferroni correction for multiple comparisons (*P*_uncorrected_ = .006, *P*_corrected_ = .072). Other connectivity parameters before or after levodopa intake did not predict emergence of LID (Supplementary Table 1).

**Table 2:** Performance of classifiers

Cortical resting‐state connectivity with putamen				
Model	Regions	*P*	Accuracy, %	AUC
RS‐1	Most affected SM_1_	<.0001	95.8	0.924
RS‐2	Less affected SM_1_	∼.1	62.5	0.646
RS‐3	SMA	>.5	29.2	0.160
RS‐4	rIFG	>.1	54.2	0.444
Volumetric structural measures				
Model	Regions	*P*	Accuracy, %	AUC
S‐1	Most affected SM_1_	>.5	3.6	0
S‐2	Less affected SM_1_	>.5	28.6	0.158
S‐3	SMA	>.5	0	0
S‐4	rIFG	>.5	0	0
S‐5	Most affected putamen	>.5	0	0
S‐6	Less affected putamen	>.1	50	0.418

Key parameters of the classification performance based on dopaminergic modulation of resting‐state connectivity and structural measures are listed in the table. AUC, area under the curve; SM_1_, primary sensorimotor cortex; rIFG, right inferior frontal gyrus.

**Figure 3:**
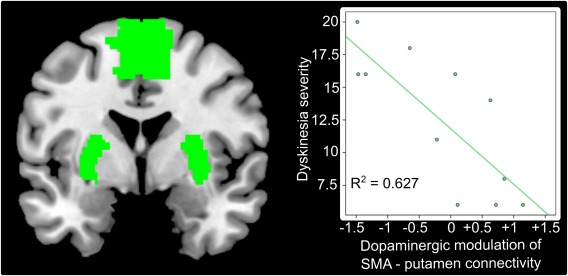
Dopaminergic modulation of resting‐state connectivity between the SMA and putamen predict dyskinesia severity (P = .004). This relationship was specific for the development of dyskinesias because dopaminergic modulation of SMA–putamen connectivity did not predict disease severity. [Color figure can be viewed in the online issue, which is available at wileyonlinelibrary.com.]

Acute dopaminergic modulation of resting‐state connectivity between the SMA and putamen predicted the severity of LID (*R* ^2^ = 0.627, *P* = .004). The more levodopa decreased resting‐state connectivity between the SMA and putamen, the more pronounced were peak‐of‐dose LID (Fig. 3). This was the case for both *z*‐scored and non‐*z*‐scored covariance estimates. Importantly, this result remained significant even when taking individual differences in the time to onset of LID into account (*R* ^2^ = 0.611, *P* = .008). There were no other significant predictions of UDysRS‐ or UPDRS‐scores (all *P*_uncorrected_ > .1). In contrast to resting‐state fMRI, regression analyses based on the volumetric MRI data failed to predict LID severity after correction for multiple comparisons.

## Discussion

We found a relation between dopaminergic modulation of cortico‐striatal resting‐state connectivity and peak‐of‐dose LID in patients with PD. First, dopaminergic modulation of resting‐state connectivity between the SM_1_ and putamen in the most affected hemisphere differentiated LID and No‐LID patients with an accuracy of approximately 95%. Second, dopaminergic modulation of SMA–putamen connectivity predicted LID severity. These results were not mediated by volumetric changes in the predefined ROIs.

The putamen is the main input structure of the basal ganglia receiving cortical glutamatergic projections, which are processed in the basal ganglia and sent back to the same cortical areas in a closed‐loop fashion. Dopaminergic afferents from substantia nigra to the putamen modulate these cortico‐striatal projections, which contribute to critical aspects of motor control such as action selection, enhancement of motor vigor, and habit formation.^26^, ^27^ In PD, nigrostriatal degeneration leads to dopaminergic deafferentation of the putamen causing impaired motor control. In line with its central pathophysiological role, previous fMRI studies consistently detected decreased motor‐related activation of the putamen in PD patients off medication, which is normalized by an intake of dopaminergic drugs.^28^ During disease progression, however, levodopa application leads to fluctuating and abnormally elevated levels of striatal synaptic dopamine because the ability to properly store and release extrinsic dopamine into the synaptic cleft is lost.^7^, ^29^, ^30^ Crucially, such elevated levels of dopamine alter the influence of cortical glutamatergic projections on striatal activity through interactions with D1‐ and D2‐type receptors,^31^, ^32^ leading to plastic changes in the cortico‐striatal synapse.^33^ This mechanism affects the processing of motor signals in the direct and indirect cortico‐basal ganglia pathways, which is thought to induce an abnormal facilitatory feedback signal to the cortical areas resulting in the emergence of LID.^9^, ^33^

In this study, abnormal dopaminergic modulation of resting‐state connectivity between the SM_1_ and putamen significantly predicted whether an individual patient would develop dyskinesias. In LID patients, resting‐state connectivity between the SM_1_ and putamen decreased, whereas it increased in No‐LID patients after levodopa intake. This was only the case for the most affected hemisphere, which is in agreement with the observation that dyskinesias first emerge on the side most affected by PD.^10^, ^34^ Importantly, classification accuracy using resting‐state SM_1_–putamen connectivity in the OFF (70.9% accuracy) or postlevodopa session (58.3%) separately was clearly inferior to classification accuracy using dopaminergic modulation of SM_1_–putamen connectivity (95.8%). This finding highlights the usefulness of using pharmacological fMRI in LID patients. It also speaks against the possibility that subclinical movements accounted for the high classification accuracy because this should also lead to high classification accuracy in the postlevodopa session where LID patients were on the verge of developing dyskinesias. Although the physiological significance of BOLD fluctuations during resting‐state is not entirely understood, it has been proposed that they encode coordination and synchronization of activity in interconnected neural areas.^3^ This suggests that levodopa intake degrades the physiological organization of the SM_1_–putamen network in LID patients. An abnormal dopaminergic modulation of SM_1_ in LID patients has also been shown with transcranial magnetic stimulation (TMS). TMS studies targeting the SM_1_ demonstrated aberrant changes in cortical plasticity after levodopa intake in LID patients^35^, ^36^ as well as an alleviating effect of M1 stimulation on LID severity.^37^, ^38^

Levodopa‐induced modulation of resting‐state connectivity between the SMA and putamen did not distinguish patients with and without LID (*P* > .5), but it predicted LID severity (*P* = .004). The more levodopa reduced resting‐state connectivity between the SMA and putamen, the more severely patients expressed peak‐of‐dose dyskinesias. This finding is in good agreement with our previous results obtained with task‐related fMRI in the same cohort.^9^, ^10^ These converging findings demonstrate the robustness of the relationship between dopaminergic modulation of SMA–putamen connectivity and LID severity across different motor states (response inhibition and rest) and analysis techniques (dynamic causal modeling and covariance analysis). The relevance of SMA in determining LID severity is further supported by TMS studies over SMA showing beneficial effects on LID severity in PD patients.^39^, ^40^ The functional role of dopaminergic modulation of cortico‐striatal networks in LID remains to be elucidated. The results of this study suggest that SM_1_‐putamen resting‐state connectivity is related to the all‐or‐nothing response to levodopa,^41^ that is, it predicts whether a patient will develop LID, whereas interindividual differences in SMA–putamen connectivity predict LID severity. Given that SMA and the adjacent pre‐SMA are centrally involved in inhibitory motor control, it is tempting to speculate that the ability of patients to control the emerging involuntary movements, as indexed by connectivity of the SMA–putamen network, determines LID severity. Such reversed inference, however, has to be met with caution and needs to be explicitly tested in future studies. At the behavioral level, one might predict levodopa‐induced deficits in inhibitory motor control in LID patients. Furthermore, interventional studies (eg, combined TMS‐fMRI) might help to disclose the causal contribution of distinct cortico‐striatal networks to the emergence of LID.

Although the rIFG is a key region for inhibitory motor control,^42^, ^43^ levodopa‐induced modulation of resting‐state connectivity between the rIFG and putamen neither differentiated patients with and without LID (*P* > .1) nor predicted LID severity. In contrast, a recent study reported that rIFG resting‐state connectivity with the putamen and SM_1_ was abnormally modulated by dopamine in LID patients using rIFG as a seed region.^11^ These findings are not necessarily in contradiction to each other because we used anatomical masks for ROI definition instead of centering ROIs on specific coordinates.^11^ Furthermore, a significant difference at the group level does not entail significant classification accuracy with respect to individual patients. Of note, one previous study reported a significant classification accuracy distinguishing patients with and without LID by measuring the structure of the right inferior frontal sulcus.^13^ Importantly, the parameter entered into the classifier was based on an already known significant group difference in the same patient group. Although such post hoc testing can be of value to illustrate group differences at the individual subject level,^10^, ^13^ it is problematic to use the same data for selection and selective analysis.^20^ In this study, we avoided this problem by using ROIs based on previously reported differences between LID and No‐LID patients rather than using specific regions based on group differences in the same resting‐state fMRI data set.

Limitations of the current study comprise the small sample size and the absence of an independent validation cohort. Furthermore, in this cross‐sectional study, patients had already developed LID. Yet it would be clinically highly relevant to assess whether a given PD patient will develop LID in the future. In contrast to task‐related fMRI, resting‐state fMRI does not require patients to engage in a specific task and can be easily standardized across imaging centers. Therefore, levodopa‐induced changes in resting‐state connectivity may serve as a powerful neuroimaging marker for emerging LID in PD. Future studies in independent validation cohorts and prospective studies are needed to test whether resting‐state fMRI can be used to identify patients at risk to develop LID. Different approaches, such as seed‐based^11^ or independent component analyses,^5^, ^44^ might both be valuable. In addition, using more well‐defined anatomical regions (eg, the foot area of the sensorimotor cortex in a patient developing LID in the foot) might further strengthen predictions of emerging LID based on resting‐state fMRI and improve the interpretability of the results. Irrespective of the exact approach used for relating resting‐state fMRI to development of LID, we suggest conducting pharmaco‐fMRI including a levodopa challenge because the neural response to levodopa encodes important information about a patient's clinical response.

## Author Roles

(1) Research Project: A. Conception, B. Organization, C. Execution; (2) Statistical Analysis: A. Design, B. Execution, C. Review and Critique; (3) Manuscript: A. Writing of the First Draft, B. Review and Critique.

D.M.H: 1A, 1B, 1C, 2A, 2B, 3A

B.N.H.: 1C, 2A, 3B

S.H.N.: 2A, 2B, 3B

K.H.M: 1B, 1C, 2A, 3B

A.L.: 1B, 1C, 2A, 3B

H.R.S.: 1A, 1B,, 2A, 3B

## Full financial disclosures for the previous 12 months

D.M.H. received funding from the European Union's Horizon 2020 research innovation programme under the Marie Sklodowska‐Curie grant agreement 655605 (which is not related to the present work). B.N.H., S.H.N., and K.H.M. have no disclosures to report. A.L. reports congress participation with Desitin and honoraria from Ipsen. H.R.S. has received honoraria as senior editor from Elsevier Publishers and research funds from Biogen‐idec not related to the present work.

## Supporting information

Additional Supporting Information may be found in the online version of this article at the publisher's web‐site.

Supplementary Information Table 1
