# Supplementary material for: Resting‐state connectivity predicts levodopa‐induced dyskinesias in Parkinson's disease
Source: Mov Disord. 2016 Mar 8;31(4):521–9. doi: 10.1002/mds.26540 (PMC5069605; doi:10.1002/mds.26540)
Supplement: Supplementary file 1 — Supplementary Information Table 1 [file MDS-31-521-s001.doc]

| **Cortical resting-state** **connectivity with putamen (OFF & ON separately)** | | | | |
| --- | --- | --- | --- | --- |
| Model | Regions | P-value | Accuracy | AUC |
| OFF-1 | most affected SM_1_ | ∼ 0.006 | 70.9 % | 0.7361 |
| OFF-2 | less affected SM_1_ | > 0.1 | 50 % | 0.4375 |
| OFF-3 | SMA | > 0.1 | 37.5 % | 0.2917 |
| OFF-4 | rIFG | > 0.5 | 0 % | 0 |
| ON-1 | most affected SM_1_ | > 0.1 | 58.3 % | 0.6181 |
| ON-2 | less affected SM_1_ | > 0.5 | 0 % | 0 |
| ON-3 | SMA | > 0.5 | 0 % | 0 |
| ON-4 | rIFG | > 0.5 | 0 % | 0 |

Key parameters of the classification performance based on resting-state connectivity in the OFF and post-levodopa scan separately are listed in the table. The uncorrected p-value of the OFF-1 classifier does not survive a conservative Bonferroni correction for the 12 classifier analyses conducted using resting-state connectivity yielding a critical P-threshold of 0.0042. AUC, area under the curve. SM1, primary sensorimotor cortex; SMA, supplementary motor area; rIFG, right inferior frontal gyrus.
